# Seasonal Variations in Microbial Community Structure and Function in the Waters Along the Yangtze-to-Huaihe Water Diversion Project According to Metagenomics

**DOI:** 10.3390/microorganisms14092016

**Published:** 2026-09-10

**Authors:** Hezhou Chen, Bohan Xu, Qidi Xie, Zhen Gu, Shaozhuang Guo, Shuqin Chen

**Affiliations:** Key Laboratory of Aqueous Environment Protection and Pollution Control of Yangtze River of Anhui Provincial Education Department, Anhui Provincial Key Laboratory of Intelligent Monitoring and Productivity Improvement of Cultivated Land, School of Energy, Environmental and Geomatics Engineering, Anqing Normal University, Anqing 246133, China; 172316@aqnu.edu.cn (H.C.);

**Keywords:** microbial community, water diversion, seasonal variations, microbial functions, driving mechanism

## Abstract

Water diversion projects can alleviate the uneven spatiotemporal distribution of water resources, but they may also impact functions of aquatic ecosystems in the waters along the route. Despite their importance, the temporal and spatial changes in multi-domain microbial community structure and function along the route remain poorly understood. Metagenomics was employed to investigate the community structure and function of bacteria, archaea, and fungi in the aquatic environments along the Yangtze-to-Huaihe water diversion project in winter and summer seasons. The results showed that seasonal variations may drive a trade-off in the species diversity of bacterial and fungal communities. Seasonal variations altered microbial communities (especially for the bacteria), and exerted a greater influence on community structure than spatial factors. Microbial community composition was more sensitive to seasonal fluctuations than functional genes. The species spatial turnover played a dominant role in shaping microbial communities (especially for winter) in both seasons. Archaea, bacteria, fungi and KEGG functional genes all exhibited a positive correlation with some environmental factors in summer but not in winter. PLS-SEM indicated that water quality and microbial composition directly significantly impacted functional genes. This study offers a theoretical basis for maintaining the stability of water ecological microorganisms in water transfer projects.

## 1. Introduction

The inter-basin water diversion projects, as an important measure for water resource allocation, have been widely adopted globally [1,2]. For instance, the United States, China, Brazil, and India have launched numerous inter-basin water transfer projects in recent years aimed at optimizing water resource allocation [3,4,5]. With the construction of water diversion projects, there is growing concern regarding their impacts on aquatic ecosystems within water resource protection zones. It has been observed that the extensive engineering measures involved in water diversion projects can enhance inter-basin connectivity, thereby causing significant alterations in hydrological conditions, eliminating biogeographical barriers, and compromising ecosystem integrity. Such changes may disrupt ecosystem functions, ultimately threatening the health of aquatic ecosystems [6]. Therefore, to ensure the sustainable implementation of these projects, it is imperative to gain a deep understanding of their effects on the river and lake ecosystems involved.

Microbes play a bridging role between ecology and chemistry, and undertake various ecological functions [7]. Microbes typically influence organic matter decomposition, nutrient cycling and drinking water quality, serving as key indicators of aquatic environmental health [8]. In aquatic ecosystems, the composition and metabolic functions of diverse microorganisms—including bacteria, archaea, and fungi—are shaped by a variety of factors, such as environmental filtering, spatiotemporal variations, microbial interactions, geographic location, and anthropogenic activities [6,9]. A previous study has found that the water diversion project from the Yangtze River to Lake Taihu not only modified the lake’s trophic status but also altered microbial community structures and their associated functional characteristics [10]. The spatial variability in bacterial communities in Lake Dongping reduced in the South-to-North Water Diversion Project (SNWDP), which may increase the risk of biotic homogenization among different rivers and lakes [11]. Water diversion projects can increase bacterial diversity in canals and alter the composition of phytoplankton, particularly those belonging to Cyanobacteria and Euglenozoa [12,13]. The enhanced habitat connectivity caused by the eastern route of SNWDP (ER-SNWDP) often promotes microbial homogenization and reduces ecological heterogeneity [12]. Additionally, microbial community succession can reflect changes in water quality parameters, and bacterial distribution is highly correlated with local water quality. These critical roles make microorganisms important indicators for assessing aquatic environments [14]. Therefore, comprehensively understanding the profound impacts of large-scale water diversion projects on the structural and metabolic functions of aquatic microbial communities in connected natural rivers and lakes is crucial for maintaining long-term microbial safety and ecological stability in these aquatic environments. Furthermore, implementing systematic and continuous ecological monitoring of these dynamic microbial communities facilitates the accurate assessment of water quality and the science-based management of freshwater ecosystems, ultimately ensuring the sustainable operation of these critical water resource allocation systems.

In recent years, an increasing number of studies have revealed the ecological impact of water diversion project operation on microbial communities. For example, Wei et al. utilized high-throughput sequencing to systematically track the community dynamics and assembly processes of microeukaryotes along the ER-SNWDP [15]. Moreover, an in-depth investigation was conducted to elucidate how environmental variables and water diversion jointly drive the spatiotemporal dynamics of various microorganisms along the ER-SNWDP [6]. Environmental DNA sequencing was applied by Liu et al. to assess the spatiotemporal variations in bacterial communities along the Middle Route of the SNWDP across summer and winter seasons [16]. Bacteria, fungi, protists, and metazoa were investigated as to the bacterial 16S rRNA gene and eukaryotic 18S rRNA gene along the main and northern branches of the Yellow River Diversion Project [1]. However, to date, few studies have employed metagenomics to investigate the spatiotemporal variations in microbial structure and function along water diversion projects. Our current understanding of the composition, assembly patterns, and functional genes of archaea, bacteria, and fungi in these engineered systems remains insufficient. In particular, for the YHDP project, the existing literature has only reported the impacts of the Yangtze-Huaihe Water Diversion Project (YHDP) on the bacterial community composition, phytoplankton community structure, zooplankton community assembly mechanisms, and the habitat availability for waterbirds in Lake Caizi [2,17,18,19].

Unlike other large-scale water diversion projects, the Anhui section of the Yangtze-Huaihe Diversion Project (YHDP) connects the Yangtze River and Huaihe River basins, traversing numerous natural rivers and lakes with similar climates between the Jianghuai regions. Therefore, the objectives of this study are to investigate (1) the spatiotemporal variations in the structure and function of microbial communities in rivers, lakes, and canals along the engineering route following project implementation; (2) the driving factors of these spatiotemporal changes. To address these goals, we established 15 sampling sites along the waterway and collected water samples during both winter and summer. Then we employed metagenomics to investigate the dynamics of bacterial, archaeal, and fungal communities in the aquatic environments along the Anhui section of the YHDP during both winter and summer seasons. The results comprehensively revealed the changes in microbial behavior during winter and summer in YHDP. The findings will provide a scientific basis for high-level ecological dispatching and contribute to the high-quality development of ecological water conservancy.

## 2. Materials and Methods

### 2.1. Field Sampling and Measurement of Main Water Environmental Parameters

**Figure 1:**
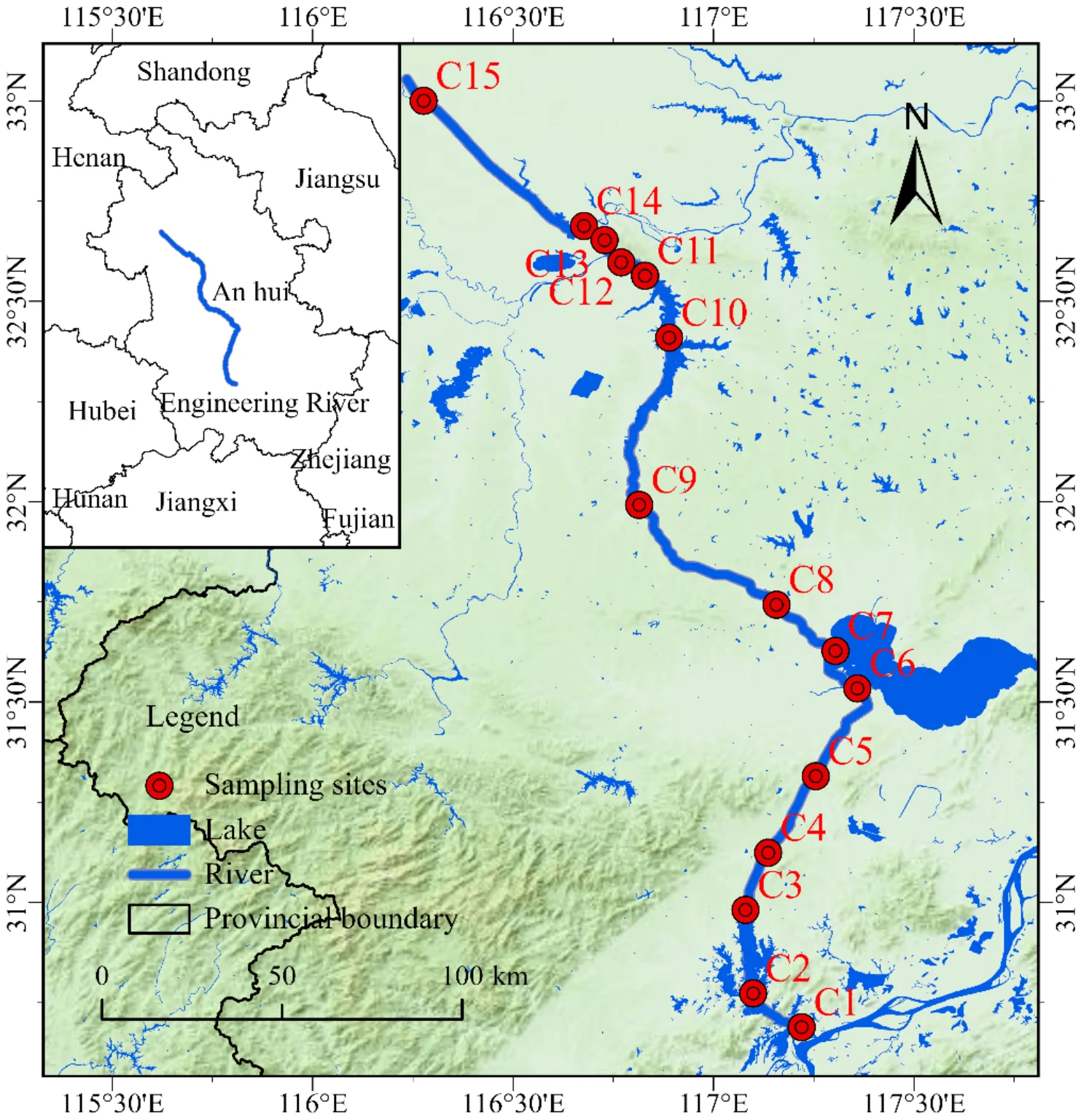
Geographical location of the Yangtze-Huaihe Diversion Project and the sampling sites.

We collected surface water samples from the same 15 sites along the YHDP in both summer and winter. Surface water sampling was conducted in January (winter) and June (summer) of 2025, yielding a total of 30 samples (Figure 1). Detailed information regarding the sampling sites is provided in Supplementary Table S1. At each site, three replicate samples were collected at a depth of 10–20 cm below the water surface and subsequently combined into a single composite sample. Volumes of 1 L and 2 L of water were collected separately for physicochemical and microbial analyses, respectively. Certain physicochemical parameters, including pH, water temperature (WT), dissolved oxygen (DO), Secchi disk depth (Z_sd_), electrical conductivity (EC), and oxidation-reduction potential (ORP) were measured in situ using a portable instrument (Aquaread, Broadstairs, UK) due to their rapid and convenient assessment. Following standard Chinese surface water testing methods, total nitrogen (TN, HJ 636-2012 in Chinese), ammonia nitrogen (NH_3_-N, HJ 535-2009 in Chinese), dissolved organic carbon (DOC, HJ 501-2009 in Chinese), permanganate index (COD_Mn_, GB 11892–89 in Chinese), and total phosphorus (TP, GB 11893-89 in Chinese) in 30 surface water samples were analyzed within 48 h [20,21]. Additionally, within 48 h of collection, approximately 2 L of water was immediately filtered through a 0.22 μm polycarbonate membrane. After filtration, a total of 30 filter membranes were obtained, and all filter membranes were stored at −80 °C for DNA extraction.

### 2.2. DNA Extraction, Metagenomic Sequencing, Metagenomic Assembly and Functional Gene Profiling

The 30 filtered membranes were transported to Shanghai on dry ice. Metagenomic shotgun sequencing libraries were constructed and sequenced by Shanghai Lingen Biotechnology Co., Ltd. (Shanghai, China). Detailed protocols for DNA extraction, metagenomic sequencing, metagenomic assembly, and functional gene analysis are provided in the Supplementary Materials.

### 2.3. Statistical Analysis

Some simple data analysis was carried out using Microsoft Excel 2024. Differences between winter and summer were investigated with independent samples *t*-test (*p* < 0.05) using IBM SPSS Statistics 27.0. The data were confirmed to meet the assumptions of normality (Shapiro–Wilk test, *p* > 0.05) and homogeneity of variance (Levene’s test, *p* > 0.05). Seasonal comparison figures of water environmental parameters, Chao1, Shannon index, Simpson index, the relative abundance (RA) of microbial taxa at the phylum level, spatial turnover and nestedness pattern indices, and niche breadth of microbial community were all drawn using Origin 2026 software.

Some statistical analyses were carried out using R 4.1.0 [22]; principal coordinates analysis (PCoA) of microorganisms and functional genes was performed using the “ape” and “ggplot2” packages in R [23]. In the resulting plots, each point represents an individual sample, with different colors or shapes indicating distinct sample groups. The proximity between points reflects their similarity and the axes represent relative distances. A permutation multivariate analysis of variance (Adonis) was performed using the “vegan” package to test the degree of significance of the differences in microbial communities and functional genes between winter and summer, and the number of permutations was 999 [24,25], and *p*-values were adjusted using the Benjamini–Hochberg false discovery rate correction. The significance threshold was set at α = 0.05. The α-diversity (observed Simpson, Shannon, Chao1) was calculated using PAST4.09. The niche breadth indices were calculated using the Levins function in the “spa” package [26]. A random forest regression model (R package randomForest) was employed to evaluate the relative importance of microbial taxa between winter and summer samples [24].

The Mantel tests via the R package “vegan” were used to examine the correlation among archaea, bacteria, fungi, KEGG functional genes and water environmental variables [27]. Partial Least Squares Structural Equation Modeling (PLS-SEM) was performed using Smart PLS 4.0 to confirm correlations between environmental factors, microbial composition, and functional genes. Prior to conducting the PLS-SEM analysis, we calculated the Variance Inflation Factor (VIF) for all predictor variables. To mitigate multicollinearity, some variables with a VIF > 5 were sequentially removed. To guarantee construct validity, variables with absolute factor loadings below 0.7 were excluded, ensuring only those meeting this criterion were included in the model. The degree of fit of this model is generally evaluated by the composite reliability (CR) values, the average variance extracted (AVE) values, the mean R^2^ value, and goodness-of-fit (GoF) index [28]. We determined the significance of the path coefficients by applying bootstrapping with 1000 iterations.

## 3. Results and Discussion

### 3.1. Temporal and Spatial Variation in Water Environmental Variables Along the YHDP Canal

**Figure 2:**
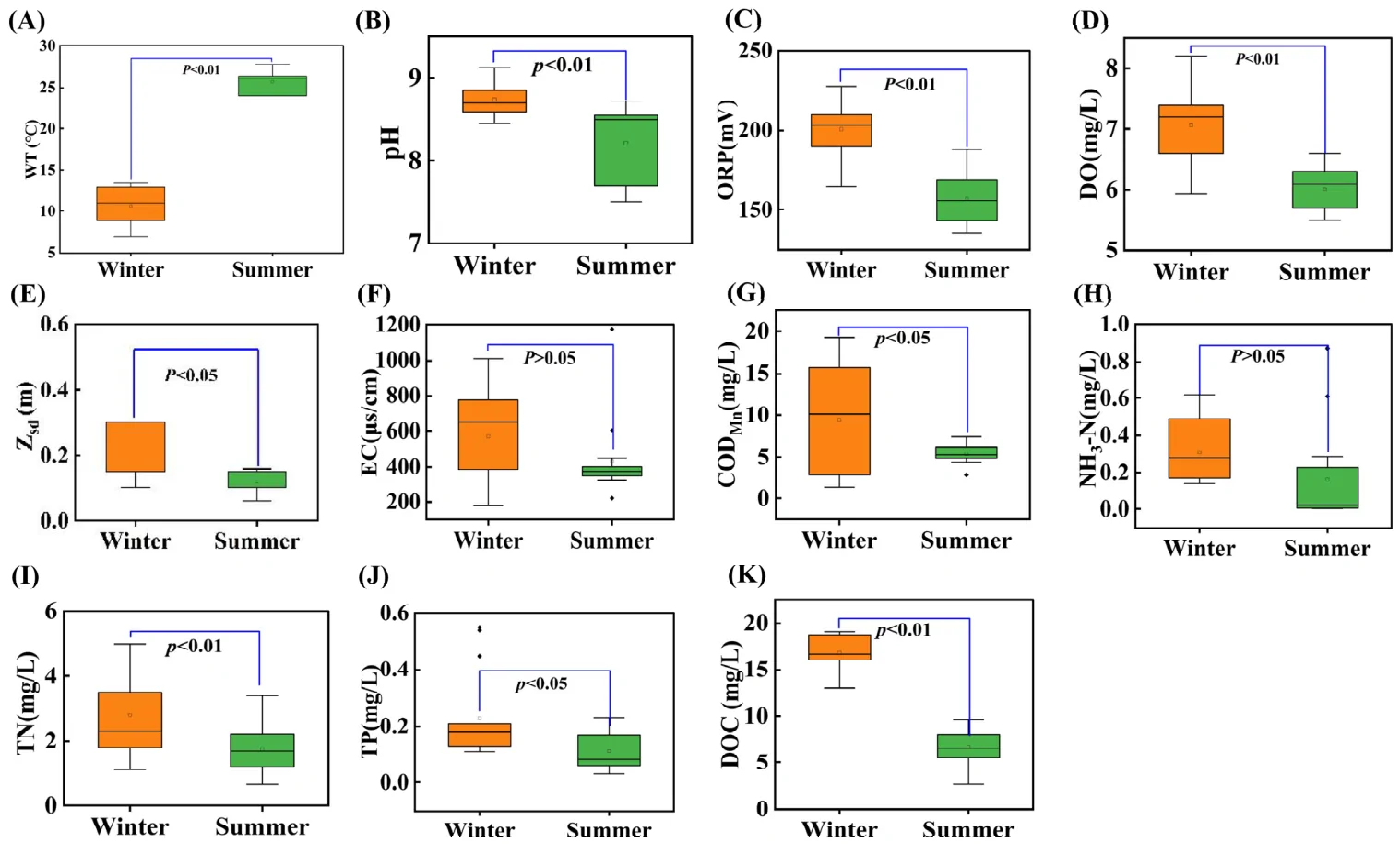
Seasonal comparison of water environmental parameters. (**A**) Water temperature (WT); (**B**) pH; (**C**) Oxidation-reduction potential (ORP); (**D**) Dissolved xygen (DO); (**E**) Secchi disk depth (Z_sd_); (**F**) Electrical conductivity (EC); (**G**) Permanganate index (COD_Mn_); (**H**) Ammonia nitrogen (NH_3_-N); (**I**) Total nitrogen (TN); (**J**) Total phosphorus (TP); (**K**) Dissolved organic carbon (DOC).

The average water temperatures (WTs) along the YHDP canal in winter and summer were 10.64 °C and 25.66 °C, respectively. Winter exhibited a higher coefficient of variation compared to summer (Figure 2, Table 1 and Figure S1). The average pH values were 8.75 in winter and 8.21 in summer. DO concentrations were 5.9–8.2 mg/L in winter and 5.5–6.6 mg/L in summer. DOC content ranged from 2.64 mg/L to 19.1 mg/L across the two seasons, with average values of 16.83 mg/L in winter and 6.72 mg/L in summer. The average EC values were 572 us/cm in winter and 432 us/cm in summer. The average ORP values were 200 mV in winter and 157 mV in summer. The average Zsd values were 0.22 m in winter and 0.11 m in summer. The mean COD_Mn_, NH_3_-N, TN and TP values were 9.43, 0.31, 2.81, 0.23 mg/L in winter, respectively. The mean COD_Mn_, NH_3_-N, TN and TP values were 5.40, 0.16, 1.75 and 0.11 in summer, respectively. In conclusion, the values of pH (*p* < 0.01), ORP (*p* < 0.01), DO (*p* < 0.01), Zsd (*p* < 0.05), COD_Mn_ (*p* < 0.05), TN (*p* < 0.01), TP (*p* < 0.05) and DOC (*p* < 0.01) decreased during summer compared to winter (*p* < 0.05) except EC (*p* > 0.05) and NH_3_-N (*p* > 0.05). A recent study also reported that the DOC concentration of the Middle Route-SNWDP was relatively low in summer [29]. However, other studies have reported findings inconsistent with our results, showing that COD_Mn_, TN, TP, and DOC were all significantly higher in the wet season than in the dry season [30]. Therefore, the reasons for this seasonal variation in water quality require further investigation. Furthermore, in terms of the coefficient of variation, the coefficient values of WT, Zsd, EC, COD_Mn_, NH_3_-N, TN, TP and DOC were high (Table 1 and Figure S1), indicating that these parameters had significant spatial differences. Only the values of pH, ORP and DO in both winter and summer were below 0.15, indicating that the spatial differences in these indicators along the YHDP canal were small. These data indicated that seasonal water diversion can affect the fluctuation of water quality concentration in the YHDP, and thereby influence the composition and function of the microbial community [6].

### 3.2. The Impact of Seasonal Variations on Diversity and Composition of Microbial Communities

**Figure 3:**
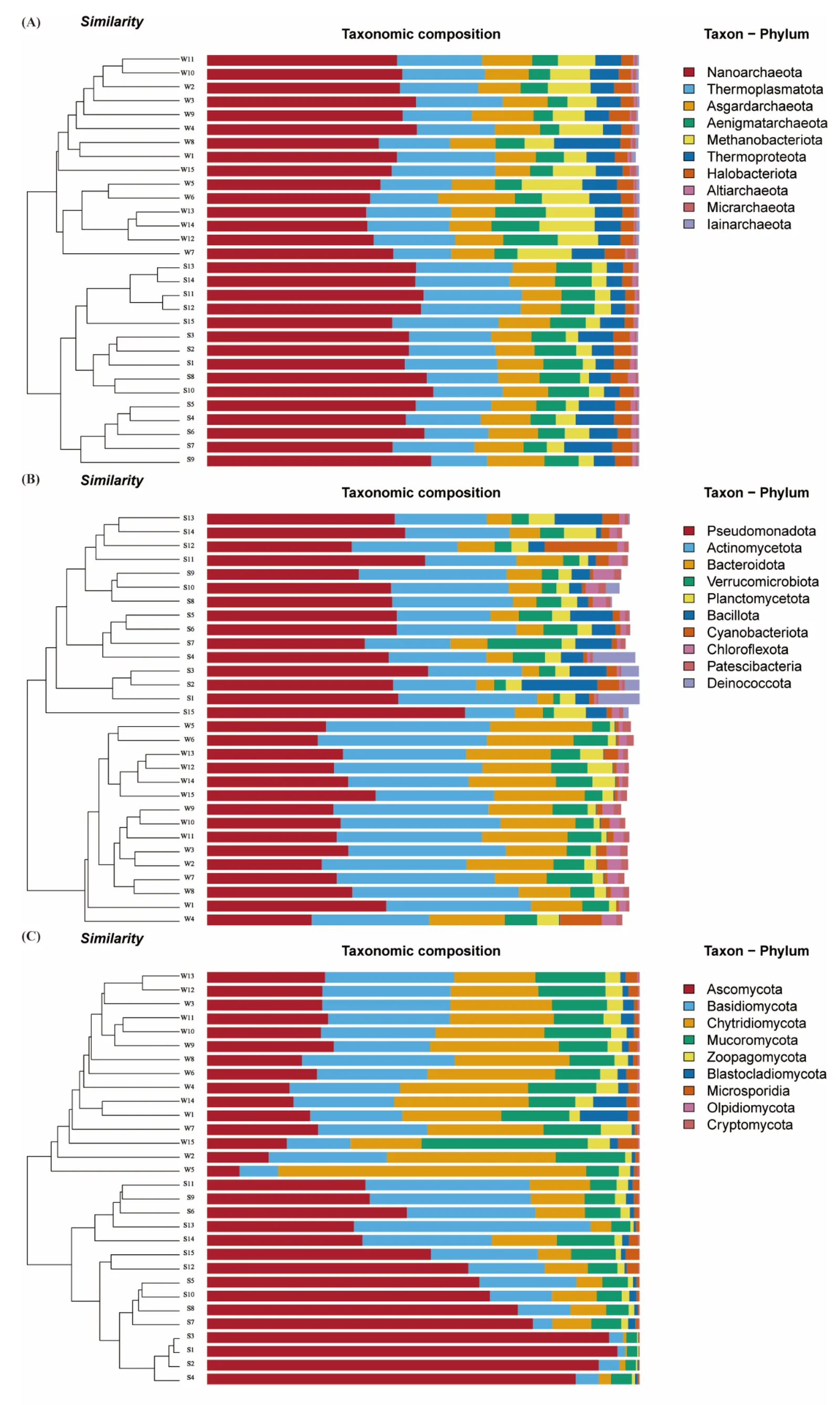
Cluster analysis of (**A**) archaeal, (**B**) bacterial and (**C**) fungal communities based on the phylum level. (Summer sample 1–Summer sample 15: S1–S15; Winter sample 1–Winter sample 15: W1–W15.)

Cluster analysis of microbial communities at the phylum level demonstrates that seasonal variation was higher than spatial variation (Figure 3). The composition of archaeal, bacterial and fungal communities all exhibited a distinct clustering effect according to the seasons. Nanoarchaeota (average relative abundance (ara) = 45.00%), Thermoplasmatota (ara = 18.79%) and Asgardarchaeota (ara = 10.68%) were common dominant phylum of archaea (Figure 3A and Figure S2A). A previous report also indicated that the archaeal communities in surface water are mainly composed of Nanoarchaeota and Thermoplasmatota [31]. For bacteria, Pseudomonadota (ara = 36.73%), Actinomycetota (ara = 28.28%) and Bacteroidota (ara = 11.76%) were common dominant phyla of bacteria (Figure 3B and Figure S2B). They are commonly reported in inland rivers [32]. Notably, the RA of Actinomycetota was the highest in winter while Pseudomonadota was the highest in summer. Ascomycota (ara = 36.73%), Basidiomycota (ara = 28.28%) and Chytridiomycota (ara = 11.76%) were common dominant phyla of fungi (Figure 3C and Figure S2C). The abundance of Ascomycota increased sharply from 22.69% in winter to 62.79% in summer, making it the most dominant phylum.

**Figure 4:**
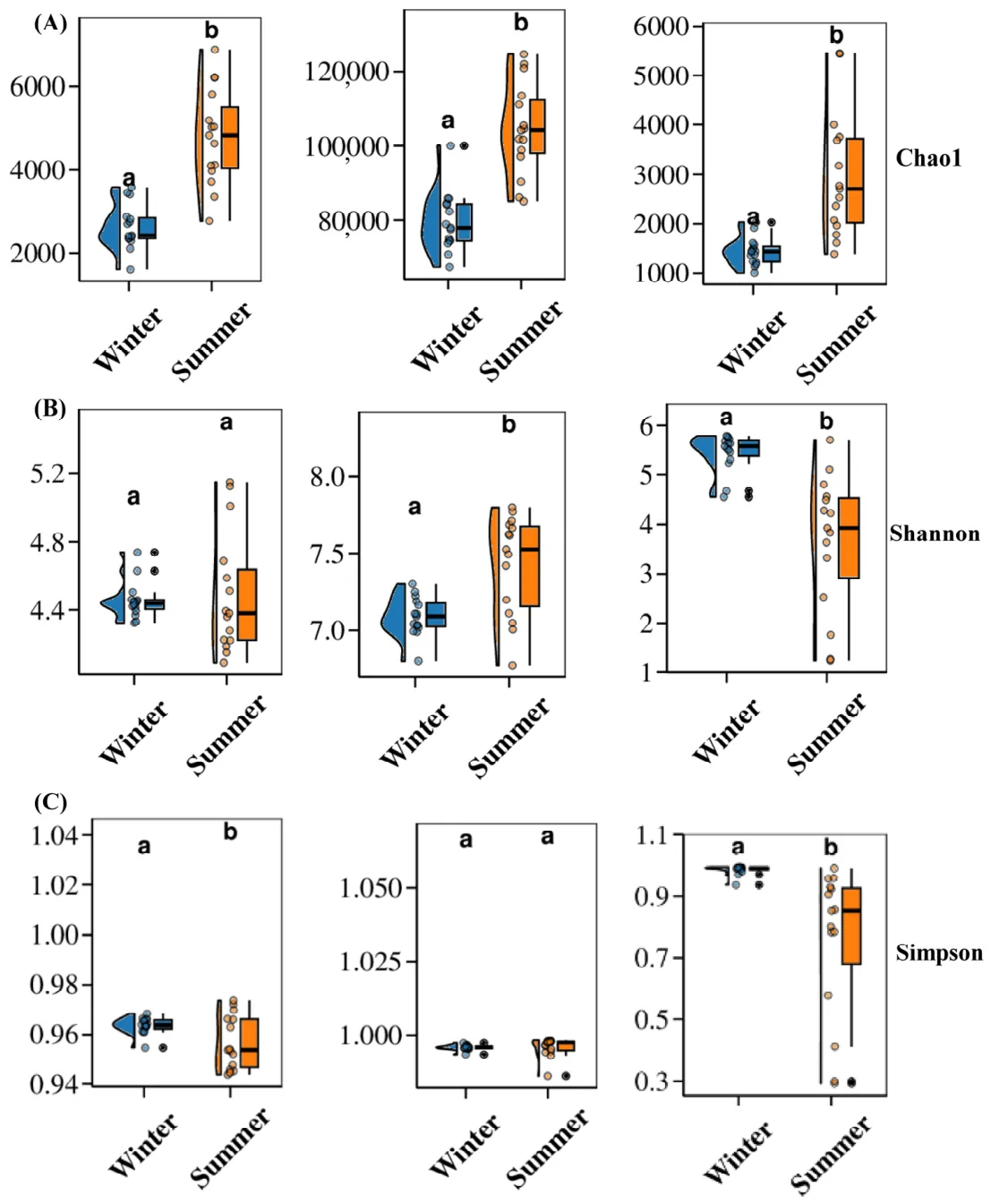
(**A**) Chao1; (**B**) Shannon index; (**C**) Simpson index during the winter and summer. (Each subfigure shows archaea, bacteria and fungi from left to right. The same letters indicate no significant difference, while different letters indicate significant difference, the same below).

It is well known that the diversity and richness of microbial communities serve as indicators of their adaptive capacity to the surrounding environment [33]. Figure 4 presents a comparative overview of α-diversity indices between winter and summer, highlighting the substantial discrepancies that were observed across the different sample groups. Archaea, bacteria, and fungi exhibited significantly higher Chao1 richness in summer compared to winter (*p* < 0.05), indicating that microbial species exhibited greater richness in summer compared to winter. The Shannon index of bacteria was higher in summer than in winter, but the opposite was observed for fungi (*p* < 0.05). This showed that seasonal variations may drive a trade-off in the species diversity of bacterial and fungal communities. This phenomenon can be attributed to the microbial selection effect. This effect is primarily driven by the movement of space or the redistribution of resources during the seasonal hydrological changes. This process can filter out certain microbial taxa, thereby facilitating the large-scale reproduction and enrichment of the well-adapted taxa [34]. The coefficient of variation of the α-diversity index at 15 sampling points in the two seasons was also calculated. The results show that the coefficients of archaea, bacteria and fungi in both seasons were all less than 0.03, indicating that the spatial differences of the α-diversity index were very small.

**Figure 5:**
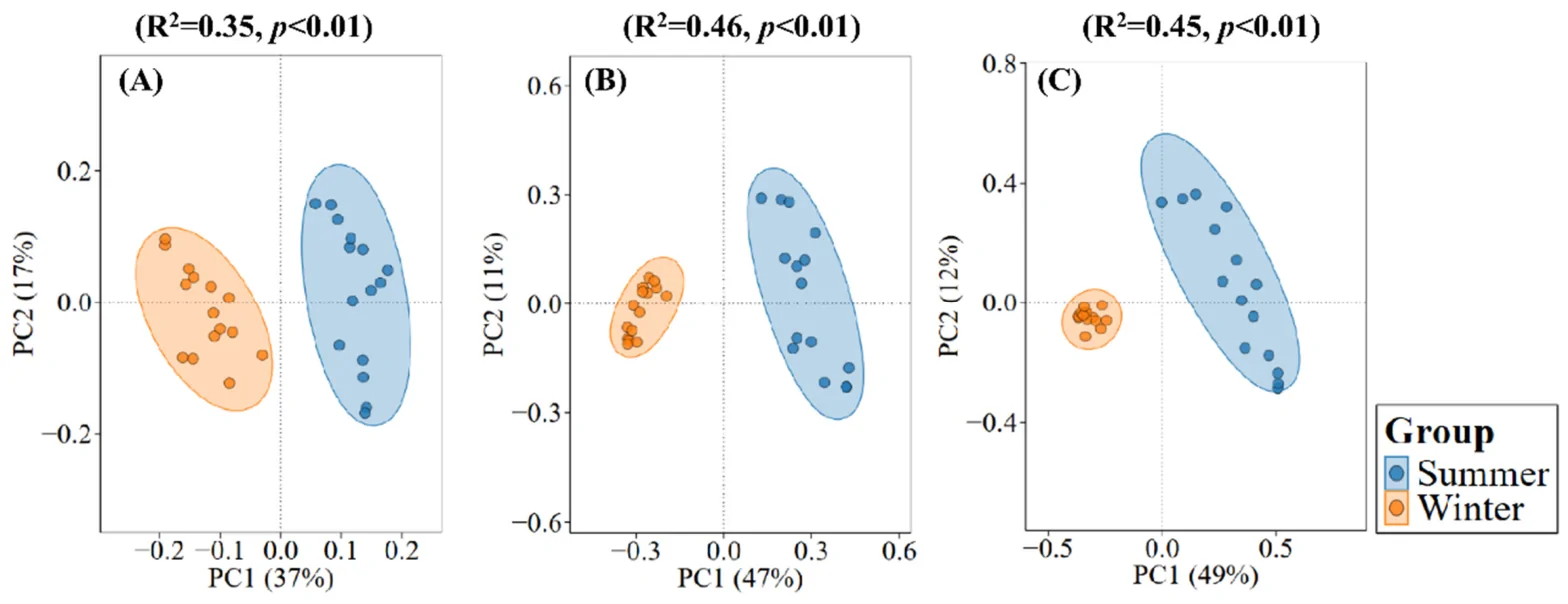
Microbial PCoA analysis and Adonis test. (**A**) archaea; (**B**) bacteria; (**C**) fungi.

PCoA analysis highlighted that there were significant variations in the composition of archaeal (R^2^ = 0.35, *p* < 0.01), bacterial (R^2^ = 0.46, *p* < 0.01) and fungal communities (R^2^ = 0.45, *p* < 0.01) in summer and winter, which indicated that seasonal variations changed the microbial community, especially for the bacteria (Figure 5). This indicated species turnover within microbial communities during the period of seasonal change [35]. Furthermore, the PCoA analysis also demonstrated that the seasonal variations in the microbial community structure along the route were greater than the spatial variations. It is consistent with the result that the seasonal differences in the composition and α-diversity of the microbial community were greater than the spatial differences. This may be attributed to the fact that the 15 sampling sites are all located within Anhui Province, sharing similar climatic backgrounds at a small spatial scale.

**Figure 6:**
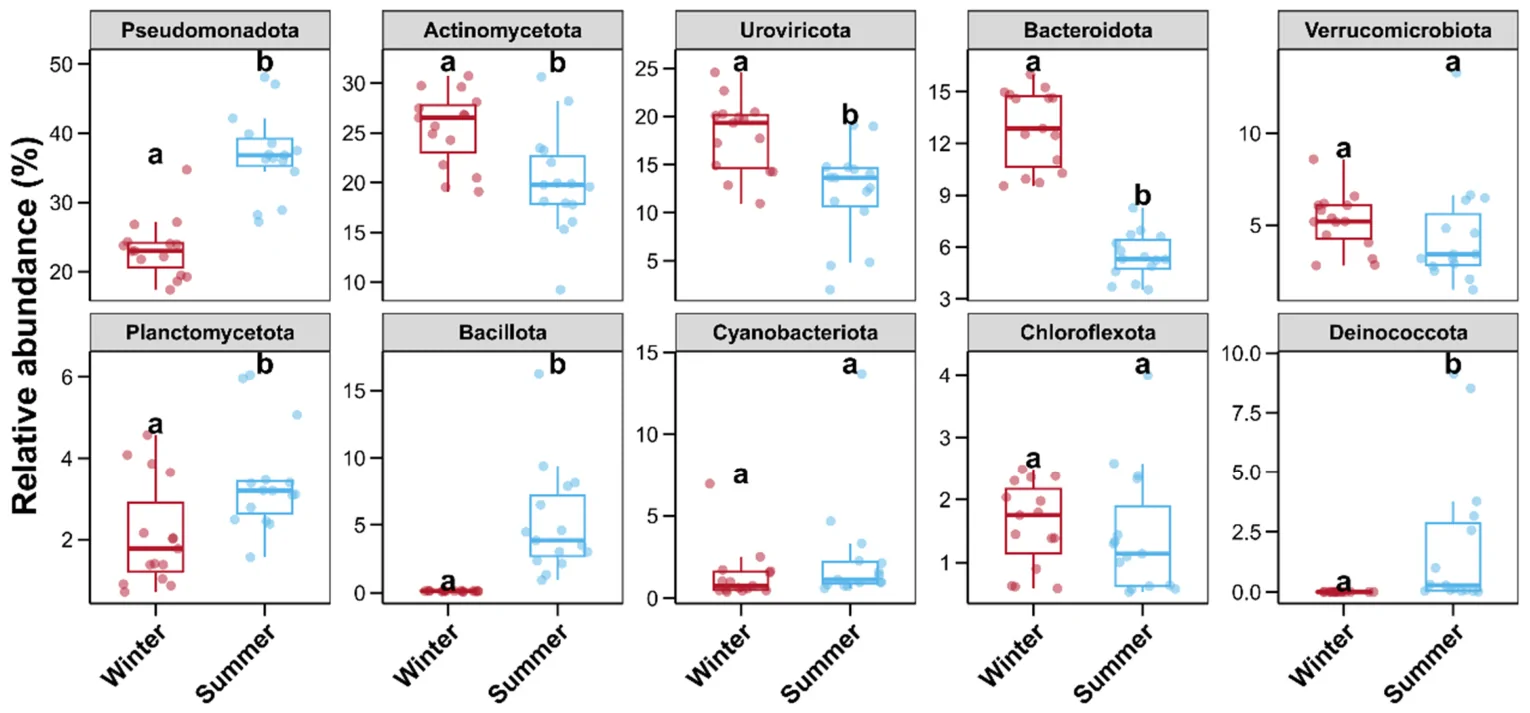
Seasonal variations in the relative abundance (RA) of microbial taxa based on the phylum level.

**Figure 7:**
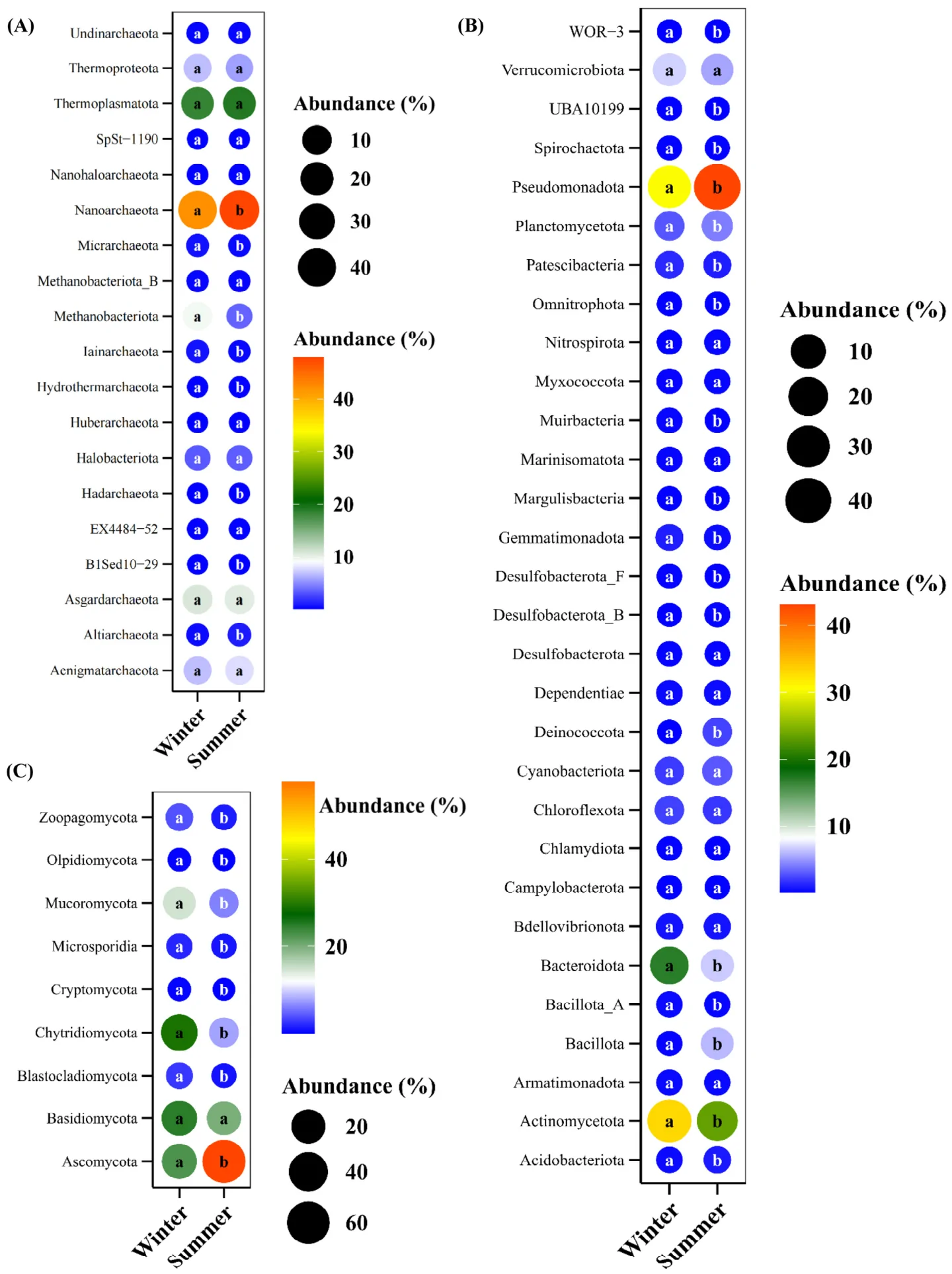
Differential bubble plots of (**A**) archaea, (**B**) bacteria and (**C**) fungi in winter and summer (based on the abundance of the top 30 phyla; if less than 30 phyla are detected, all detected species are shown).

In addition, we conducted a seasonal difference analysis on the top10 phyla in terms of RA (Figure 6). The result suggested that there was a significant increase in the relative abundances of Pseudomonadota, Planctomycetota, Bacillota, and Deinococcota in summer versus winter (*p* < 0.05). Pseudomonadota is believed to play a widespread role in various carbon and nitrogen metabolic pathways [36,37]. Deinococcota was confirmed to play a crucial role in the process of organic matter degradation [33]. Therefore, compared to winter, summer may be more conducive to the degradation of carbon and nitrogen. In contrast, the relative abundances of Actinomycetota, Uroviricota, and Bacteroidota were markedly greater in winter compared to summer (*p* < 0.05). The enriched taxa of Uroviricota, Actinomycetota and Bacteroidota in winter might be attributed to the high concentration of DOC, COD_Mn_, TN and TP which can promote microbial niche differentiation and select pollution-tolerant microbial taxa [38]. Figure 7 further reveals the effects of seasonal variations on archaea, bacteria and fungi. For archaea, a total of eight major phyla showed significant differences (e.g., Nanoarchaeota, Micrarchaeota, Methanobacteriota). The number of bacterial phyla detected was the highest, with up to 18 major phyla showing significant differences. According to the RA, they were ranked as Pseudomonadota, Actinomycetota, Bacteroidota, Bacillota, and Planctomycetota. The number of fungal phyla detected was the lowest, with eight major phyla showing significant differences (e.g., Ascomycota, Chytridiomycota, Mucoromycota). Moreover, the random forest model indicated that Desulfobacterota_B, Iainarchaeota, Bacillota, Latescibacterota, and Zoopagomycota exhibited high importance in distinguishing between the winter and summer samples (Figure S3).

### 3.3. The Influence of Seasonal Variations on the Microbial Community Assembly Patterns

**Figure 8:**
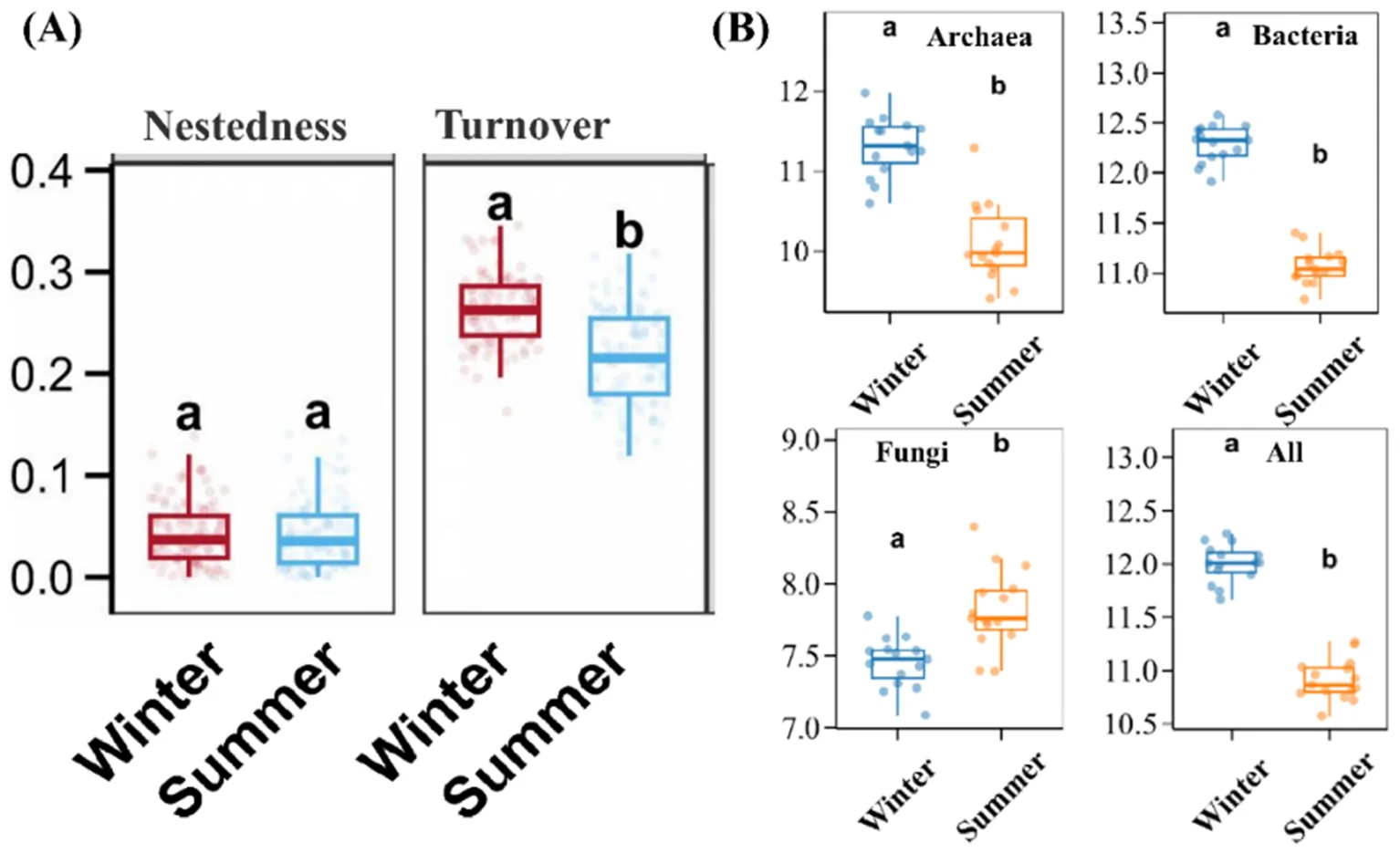
(**A**) Comparisons of spatial turnover and nestedness pattern indices among biological groups; (**B**) niche breadth of microbial community.

The Simpson dissimilarity index (β_SIM_) serves as a reliable indicator for quantifying the degree of species turnover across microbial communities, while the nestedness-resultant dissimilarity index (β_NES_) is specifically employed to reflect the species nestedness structure, two key ecological processes that fundamentally shape the biotic similarity of microbial assemblages in different seasons [39]. In this study, it was found that turnover values were much higher than nestedness values in the two seasons (Figure 8A), indicating the species spatial turnover exerted a predominant effect on the structuring of microbial communities during different hydrological periods. Similarly, it was also found that the spatial turnover of species assemblages is the primary driver shaping the summer microbial community structure in the rivers of the ER-SNWDP [40]. Furthermore, turnover values were higher in winter than in summer, suggesting that the microbial community structure in winter is more influenced by species spatial turnover than in summer. This may be attributed to the seasonal variations in environmental filtering, such as water temperature and nutrients.

The capacity of a community to adapt to environmental heterogeneity can be gauged through niche breadth, which also acts as an important indicator of species tolerance to environmental shifts [41]. In this study, compared with winter, archaeal and bacterial niche breadth decreased in summer, but fungal niche breadth instead increased (Figure 8B), showing that seasonal changes had opposite effects on the niche breadth of fungi and bacteria. This inverse trend is likely rooted in fundamental physiological differences between bacterial and fungal communities in response to seasonal fluctuations in resource availability. This might be attributed to the fact that fungi are often better adapted to degrading complex organic matter or resisting different environmental stresses than specific aquatic bacteria [42] This discovery is similar to the significant negative correlation between the niche breadth of soil bacteria and fungi [43]. Moreover, the niche breadth of bacterial communities (the average value of all samples = 11.68, the same below) was much greater than that of fungal communities (7.64). Overall, the niche breadth of microorganisms during summer (10.91) was lower than that during winter (12.00). This indicated that the total bioavailable resources in the water decreased during winter, prompting microorganisms to expand their niches to widen the area of available resources [44]. We also acknowledge the limitations of this dataset in directly resolving community assembly mechanisms. Specifically, the single-year sampling fails to capture interannual fluctuations and no spatial replicates were included at the sampling sites. In future studies, we plan to combine long-term time-series sampling with comprehensive monitoring of multiple environmental factors to further elucidate the driving mechanisms of seasonal variations in community assembly.

### 3.4. The Impact of Seasonal Variations on the Microbial Functions

**Figure 9:**
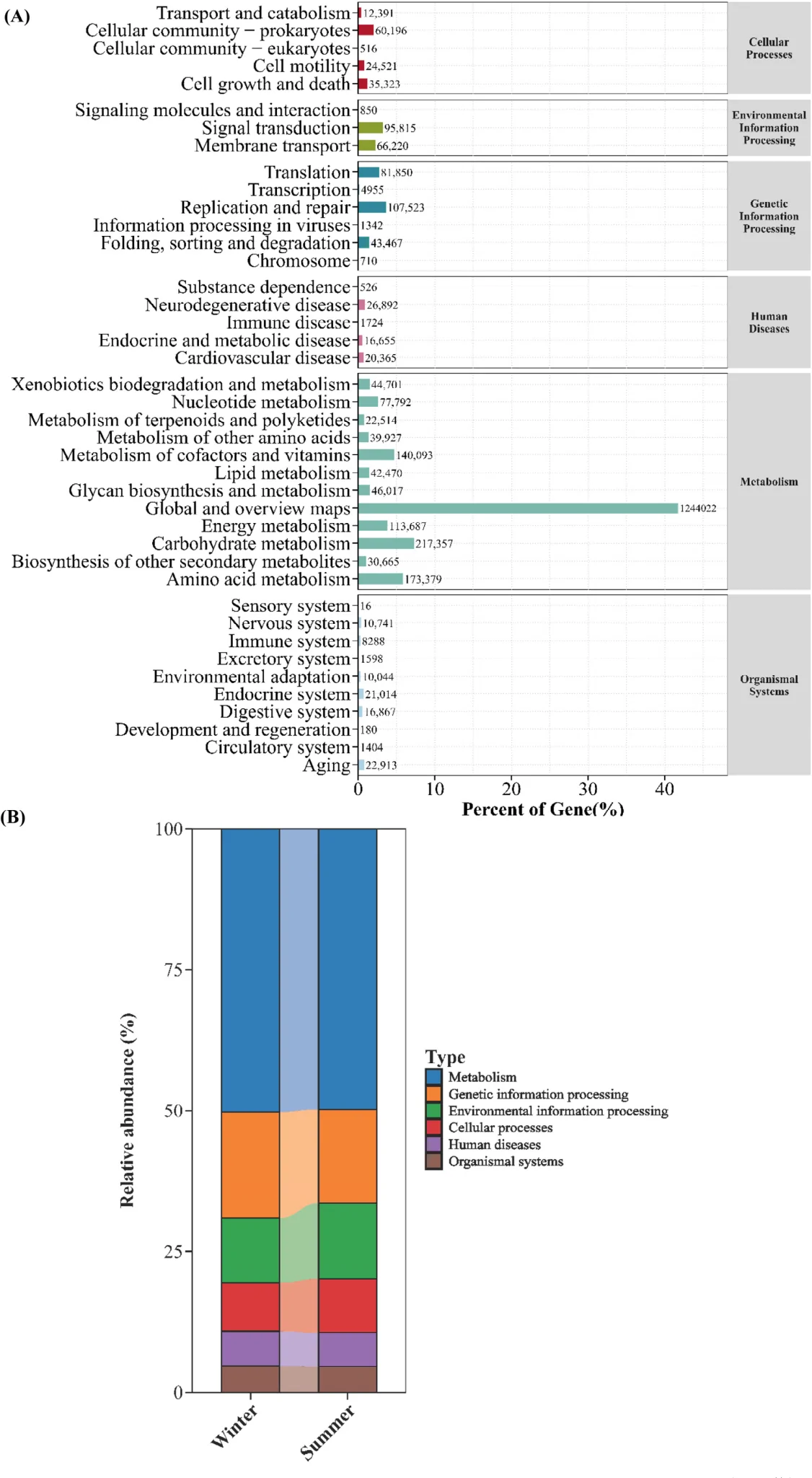
(**A**) Six types of KEGG metabolic pathways; (**B**) seasonal comparison of six types of KEGG metabolic pathways.

A comprehensive functional comparison utilizing the KEGG database demonstrated that, among all level 1 pathway categories, metabolism contained the most abundant number of annotated genes (Figure 9A). Based on the KEGG database classification, the annotated genes were distributed across six functional categories. In terms of RA, these pathways were ranked in descending order as: metabolism, genetic information processing (GIP), environmental information processing (EIP), cellular processes (CP), human diseases (HD), and organismal systems (OS) (Figure 9B). This indicates that microbes in both winter and summer participated in carbon, nitrogen, and phosphorus cycling through metabolic processes such as assimilation and dissimilation, thereby promoting nutrient transformation and organic matter degradation. Microbial metabolism played a dominant role [45]. The RA of EIP decreased significantly in winter relative to summer (*p* < 0.05); conversely, GIP was significantly elevated in winter (*p* < 0.05). This indicated that some functional genes of water bodies in different seasons along the YHDP perform different functions in a coordinated manner during the process of adaptation to environmental stress [46].

To compare the temporal and spatial differences in the composition and functional genes of microbial communities between winter and summer, we used PCoA analysis again. The result showed significant differences in microbial communities (R^2^ = 0.43, *p* < 0.01) and functional genes (R^2^ = 0.36, *p* < 0.01) between winter and summer (Figure S4), indicating that seasonal transform had a greater effect on the composition of microbial communities than on functional genes. This can be explained by the theory that microbial community structure determines function [47]. Furthermore, like microbial communities (Figure 8B), functional gene niche breadth was also elevated in winter (18.94) relative to summer (18.07) (Figure S5). Nevertheless, the niche breadth of microbial communities exhibited greater sensitivity to seasonal variations compared to that of functional genes.

### 3.5. The Correlations Among Microbial Communities, KEGG Functional Genes, and Environmental Factors

**Figure 10:**
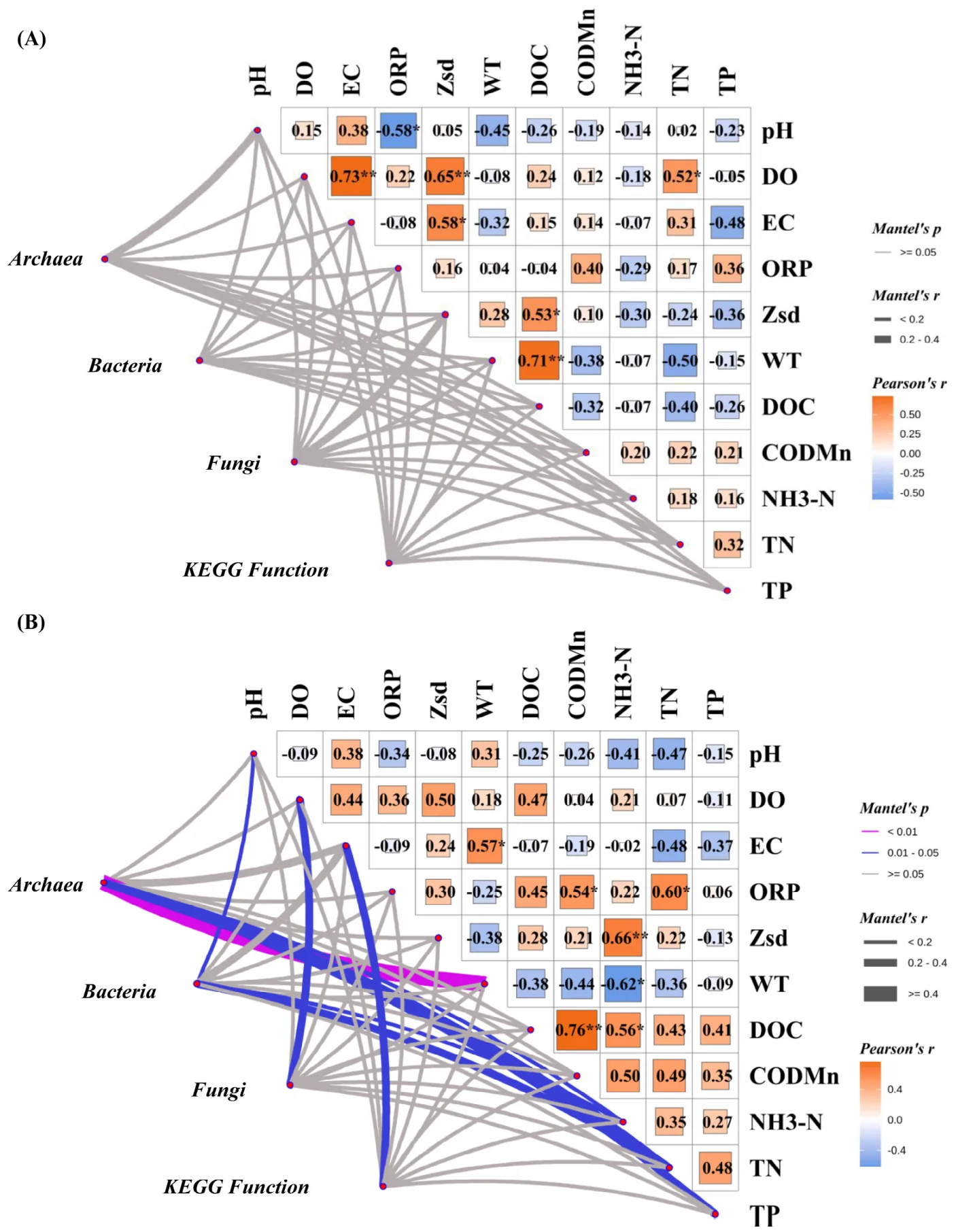
Mantel test of physical and chemical parameters, and water quality with microbial community and KEGG functional genes. (**A**) in winter; (**B**) in summer. (∗ and ∗∗ indicate whether the correlation is significant. ∗ represents *p* < 0.05, and ∗∗ represents *p* < 0.01).

To elucidate the complex relationships between the surrounding habitat and microbial ecology, Mantel tests were employed to systematically evaluate the impact of various environmental factors on archaeal (top 10 phyla), bacterial (top 10 phyla), fungal community (9 phyla) and KEGG functional genes during the winter and summer. The result revealed that archaea, bacteria, fungi and KEGG functional genes all exhibited a positive correlation with some environmental factors in summer but not in winter (Figure 10). This might be attributed to the low water temperature in winter, which reduces metabolic capacity [29,48]. Specifically, the archaeal community presented a significant positive correlation with WT (correlation coefficient r = 0.58 and *p* < 0.01; the same below), NH_3_-N (r = 0.19 and *p* < 0.05), TN (r = 0.27 and *p* < 0.05) and TP (r = 0.24 and *p* < 0.01). The bacterial community presented a significant positive correlation with pH (r = 0.19 and *p* < 0.05), NH_3_-N (r = 0.16 and *p* < 0.05), and TN (r = 0.36 and *p* < 0.05). The fungal community only presented a significant positive correlation with DO (r = 0.25 and *p* < 0.05). KEGG functional genes only displayed a significant positive correlation with EC (r = 0.35 and *p* < 0.05). These data indicated that archaea are likely to be more susceptible to the influence of WT than bacteria and fungi. In general, the community composition of archaea and bacteria was more strongly shaped by environmental factors compared to fungi and KEGG functional genes. Relevant studies have shown that pH, DO, WT and water nutrients also significantly impact microorganisms in the water [49,50]. The present findings underscored the profound impact of summer environmental fluctuations on both the structural composition of microbial communities and their underlying KEGG functional profiles in YHDP.

**Figure 11:**
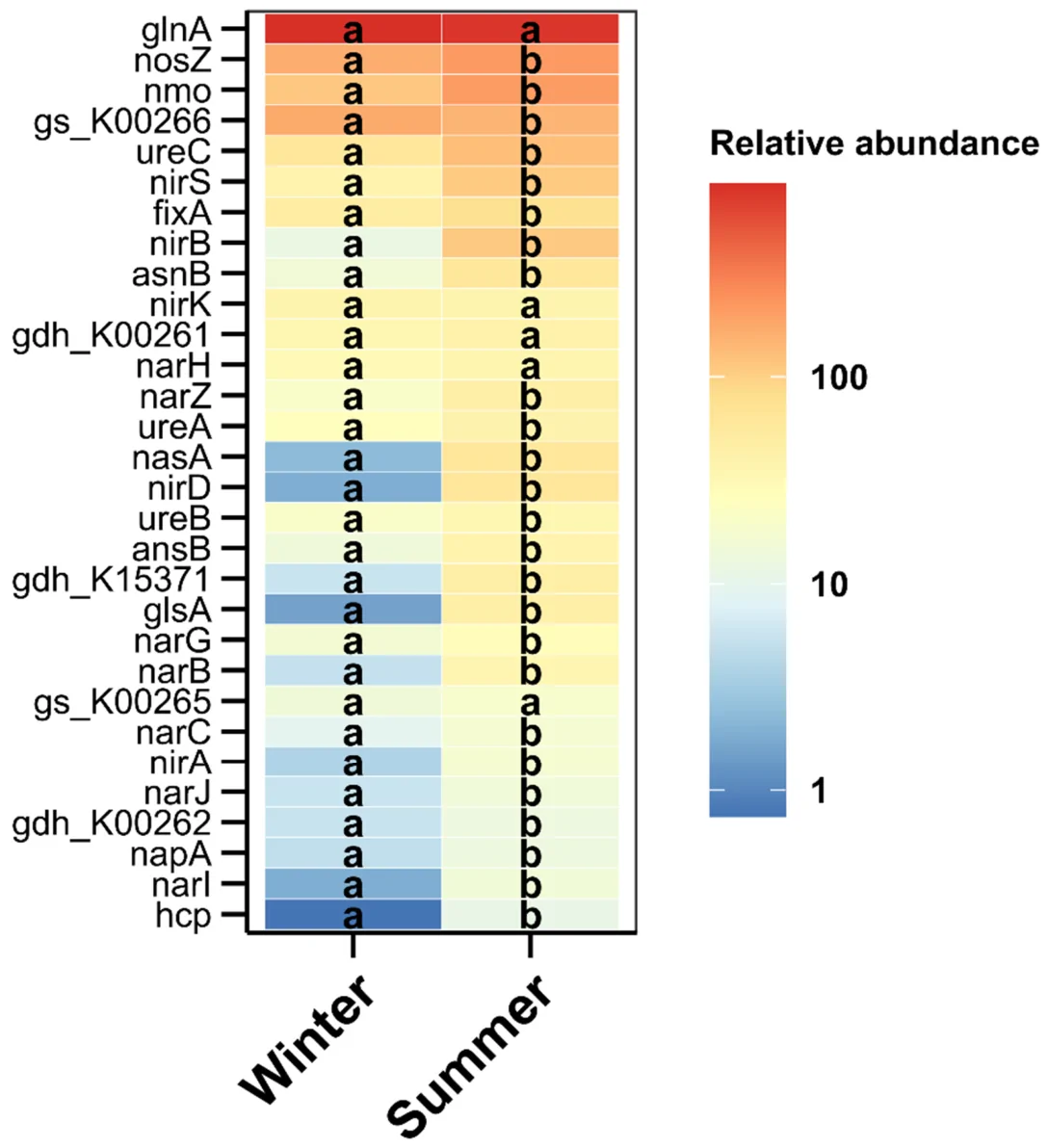
Heatmap of genetic differences in nitrogen cycling between summer and winter.

Given the strong correlation between total nitrogen and the composition of the microbial community, we further analyzed the differences in the RA of genes related to the nitrogen cycle in the two seasons (Figure 11). The result indicated that the relative abundances of nosZ, nmo, ureC, nirS, fixA, nirB, asnB, narZ, ureA, nasA and nirD were significantly higher in summer than in winter. nosZ, nirS, and narZ are involved in the denitrification process [51,52]. nasA, nirB, and nirD participate in the assimilation/disassimilation nitrate reduction process [53]. nmo, ureC, ureA, asnB are all involved in the process of organic nitrogen metabolism [54]. fixA has the function of nitrogen fixation. Therefore, this result demonstrated that the nitrogen cycle in water bodies of YHDP may be more active in summer than in winter. These results also elucidated the close correlation between the microbial community and TN and NH_3_-N in summer (Figure 10B).

**Figure 12:**
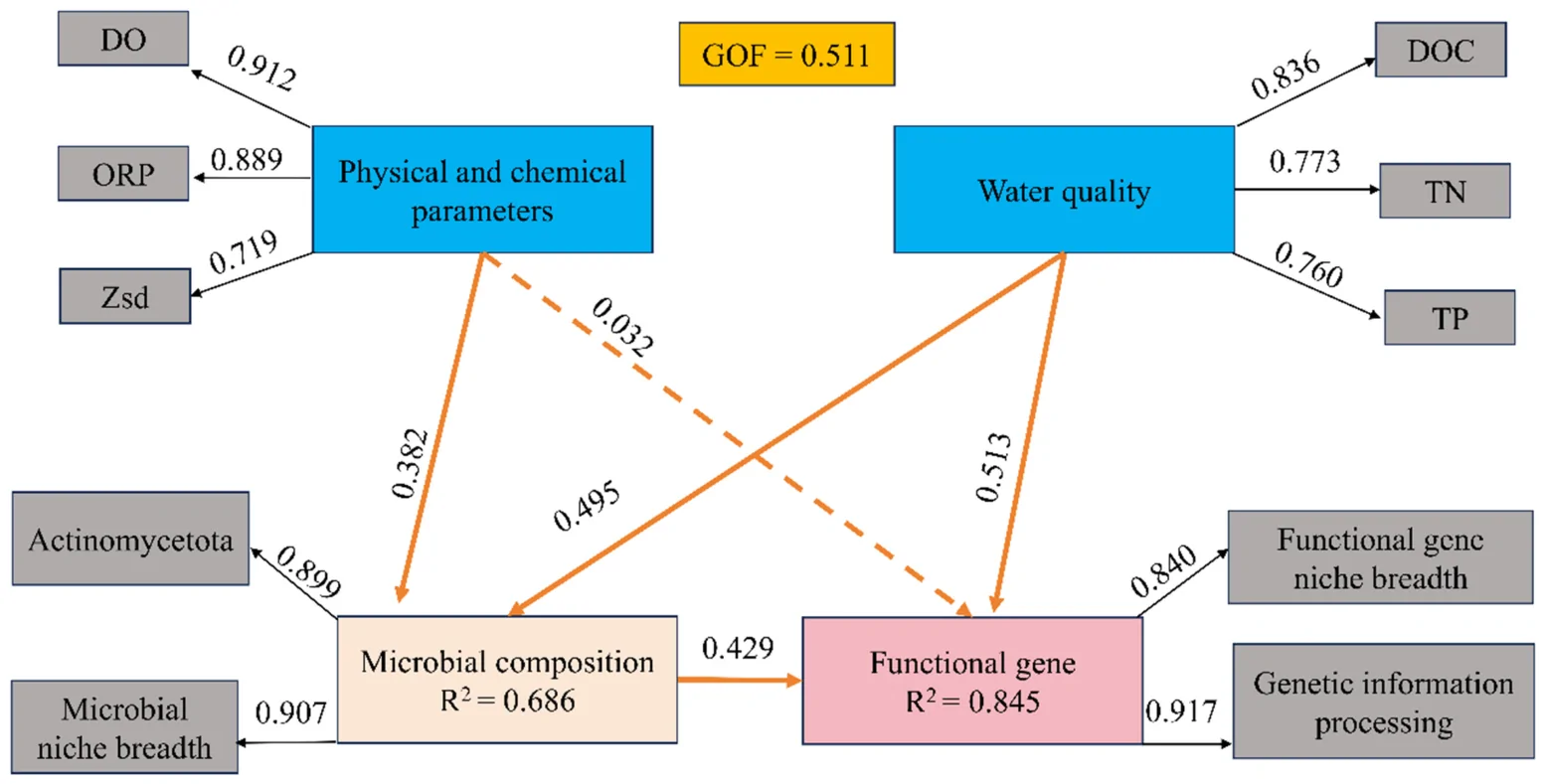
The influence mechanism of environmental factors on the composition and function of microbial communities based on PLS-SEM. (The solid arrows represent statistically significant relationships, while the dashed arrows represent statistically insignificant relationships. GOF, goodness-of-fit index.)

Furthermore, to further elucidate these interactions, PLS-SEM was used to reveal the influence mechanism of environmental factors on the composition and function of microbial communities. Broadly speaking, water quality parameters also fall under the category of physical and chemical parameters. However, water quality parameters describe the standing stock of carbon, nitrogen and phosphorus substrates that supply material and energy for microbial catabolism and anabolism. This study was conducted to further analyze the impact of water quality parameters on microorganisms. Therefore, the water quality parameters were singled out for PLS-SEM study. After adjusting and testing the model several times, we constructed the PLS-SEM using physical and chemical parameters (DO, ORP, Z_sd_), water quality (DOC, TN, TP), microbial composition (the RA of Actinomycetota, microbial niche breadth), and functional gene (functional gene niche breadth, the RA of genetic information processing) (Figure 12). It is found that the CR values are greater than 0.7, and the AVE values are higher than 0.5 in this study (Table S2), indicating that all constructs have sufficient convergent validity [55]. The AVE value of functional gene is greater than that of water quality, indicating that the measurement error of functional gene is smaller. The mean R^2^ value was 0.745, indicating that the PLS-SEM has moderate prediction accuracy [56]. The model’s goodness-of-fit index is 0.511, indicating that the model effectively explains the influence mechanism [57]. The physical and chemical parameters and water quality directly significantly influence microbial composition, with impact coefficients of 0.382 (*p* < 0.05) and 0.495 (*p* < 0.05), respectively. Water quality and microbial composition directly significantly impact functional gene, with a direct impact coefficient of 0.513 (*p* < 0.05) and 0.429 (*p* < 0.05). However, the physical and chemical parameters had no direct significant influence on the functional genes (*p* > 0.05). This might be because the concentrations of carbon, nitrogen and phosphorus directly exerted driving pressure on the functional genes. Because the R^2^ value for the latent variable representing functional gene was 0.845 in our study, it can be deduced that physical and chemical parameters, water quality and microbial composition were responsible for explaining 84.5% of the variance in functional gene. In agreement with the present study, previous studies have also demonstrated that microbial communities can respond to environmental changes, rapidly adjusting their structure and function through growth, migration, and adaptation [58,59]. Furthermore, combined with the results of the Mantel test (Figure 10), we can infer that the composition and function of microbial community are more impacted by environmental factors in summer than in winter.

## 4. Conclusions

Comprehending the seasonal dynamics of the composition and functions of archaeal, bacterial, and fungal communities is of paramount importance for the adaptive management of the YHDP in China. This study revealed that microbial species exhibited greater richness in summer compared to winter. Seasonal variations may drive a trade-off in the species diversity of bacterial and fungal communities. Seasonal variations changed the microbial communities, particularly bacterial communities, and exerted a greater influence on community structure than spatial factors along the route. Furthermore, functional genes were less affected by seasonal changes, while the composition of microbial communities was more sensitive. The structuring of microbial communities in winter and summer was predominantly governed by species spatial turnover. Archaea, bacteria, fungi and KEGG functional genes all exhibited a positive correlation with some environmental factors in summer but not in winter. The community composition of archaea and bacteria in summer was more strongly shaped by environmental factors compared to fungi and KEGG functional genes. PLS-SEM indicated that water quality and microbial composition directly significantly impacted functional gene, with a direct impact coefficient of 0.513 (*p* < 0.05) and 0.429 (*p* < 0.05), but the physical and chemical parameters had no direct significant influence on the functional genes (*p* > 0.05). The composition and function of microbial community were more affected by environmental factors in summer than in winter. These findings elucidate the dynamic shifts in microbial communities across seasonal hydrological cycles, underscoring the combined role of seasonal variations and water environments in driving microbial succession.

However, this study prioritizes assessing the global seasonal and spatial patterns across the entire water conveyance corridor, rather than the seasonal variations within individual sites, which constitutes a limitation of this research. The “replicates” are seasonal/spatial composites rather than true independent biological/sequencing replicates per site, which slightly reduces the capacity to evaluate intra-site variance. In future research work, we will do our utmost to ensure the repetition of the sampled samples.

## Figures and Tables

**Table 1:** The coefficient of variation in water environmental parameters in winter and summer.

Parameters	Winter	Summer
WT (°C)	0.20	0.04
pH	0.02	0.05
ORP (mV)	0.07	0.10
DO (mg/L)	0.08	0.06
Zsd (m)	0.72	0.25
EC (μs/cm)	0.44	0.49
COD_Mn_ (mg/L)	0.65	0.19
NH_3_−N (mg/L)	0.52	1.53
TN (mg/L)	0.42	0.42
TP (mg/L)	0.64	0.57
DOC (mg/L)	0.10	0.27

## Data Availability

The original contributions presented in this study are included in the article/Supplementary Material. Further inquiries can be directed to the corresponding authors.

## Supplementary Materials

The following supporting information can be downloaded at: https://www.mdpi.com/article/10.3390/microorganisms14092016/s1, Figure S1: Physical and chemical parameters at each sampling point in winter and summer. (A) WT; (B) pH; (C) ORP; (D) DO; (E) Zsd; (F) EC; (G) CODMn; (H) NH3-N; (I) TN; (J) TP; (K) DOC; Figure S2: The relative abundance of the main phyla of (A) archaea, (B) bacteria and (C) fungi; Figure S3: Random forest analysis (Total taxa - Phylum level); Figure S4: (A) Microbial communities and (B) functional gene PCoA analysis and Adonis test; Figure S5: Niche breadth of functional genes; Table S1: The detailed information of sample sites; Table S2: The CR and AVE values. References [60,61] are cited in the Supplementary Materials.
