# Supplementary material for: Seasonal Variations in Microbial Community Structure and Function in the Waters Along the Yangtze-to-Huaihe Water Diversion Project According to Metagenomics
Source: Microorganisms. 2026 Sep 10;14(9):2016. doi: 10.3390/microorganisms14092016 (PMC13609500; doi:10.3390/microorganisms14092016)
Supplement: Supplementary file 1 [file microorganisms-14-02016-s001.zip › microorganisms-4512974-supplementary.pdf]

## Text S1. DNA extraction, metagenomic sequencing, metagenomic assembly and functional gene profiling

Total genomic DNA in this study was extracted independently from membrane filters using the FastDNA™ Spin Kit for Soil (MP Biomedicals, USA) [36]. The specific operation was carried out according to the manufacturer’s instructions. A NanoDrop One spectrophotometer (Thermo Fisher Scientific, USA) was utilized to assess DNA concentration and purity, while DNA integrity was verified via 1.0% agarose gel electrophoresis. Metagenomic shotgun sequencing libraries were constructed and sequenced by Shanghai Lingen Biotechnology Co. Ltd (Shanghai, China). 30 samples in winter and summer were sequenced on an Illumina NovaSeq 6000 platform in paired-end 150 bp (PE150) strategy. Each sequencing library was derived from a unique biological sample (30 samples). Preprocessing of the raw reads was performed using fastp, which involved the removal of adapters, trimming of low-quality bases, and exclusion of short reads. After quality control of the obtained sequencing data, comprehensive metagenomic analyses were conducted using bioinformatics approaches.

The quality-filtered clean data were assembled using Megahit (https://github.com/voutcn/megahit) with a succinct de Bruijn graph (SdBG) [60]. Contigs were generated for each individual sample, and the assembly quality was evaluated by calculating N50 and N90 statistics. For functional annotation based on the Non-Redundant Protein Sequence Database (NR), predicted genes were annotated with taxonomic information. The abundance of each species was calculated by summing the gene abundances of its corresponding genes. Subsequently, species abundances were quantified across all taxonomic (Domain, Kingdom, Phylum, Class, Order, Family, Genus, and Species) ranks to construct abundance profiles for each sample. Functional annotation of the predicted genes was performed using the Kyoto Encyclopedia of Genes and Genomes (KEGG) using eggNOG-mapper (v1.0.3) [61].

Table. S1 The detailed information of sample sites

| sample | longitude | latitude |
|---|---|---|
| C1 | 117.219686 | 30.688656 |
| C2 | 117.098804 | 30.77296 |
| C3 | 117.079606 | 30.981337 |
| C4 | 117.136206 | 31.123989 |
| C5 | 117.255004 | 31.315118 |
| C6 | 117.358765 | 31.534058 |
| C7 | 117.304068 | 31.627625 |
| C8 | 117.157051 | 31.742785 |
| C9 | 116.814369 | 31.991872 |
| C10 | 116.889191 | 32.409146 |
| C11 | 116.829082 | 32.563713 |
| C12 | 116.769642 | 32.597134 |
| C13 | 116.728033 | 32.652614 |
| C14 | 116.676674 | 32.687788 |
| C15 | 116.276705 | 33.00025 |

Table. S2 The CR and AVE values

| | **composite reliability (CR)** | **average variance extracted (AVE)** |
|---|---|---|
| Physical and chemical parameters | 0.809 | 0.713 |
| Water quality | 0.707 | 0.625 |
| Microbial composition | 0.712 | 0.773 |
| Functional gene | 0.774 | 0.816 |

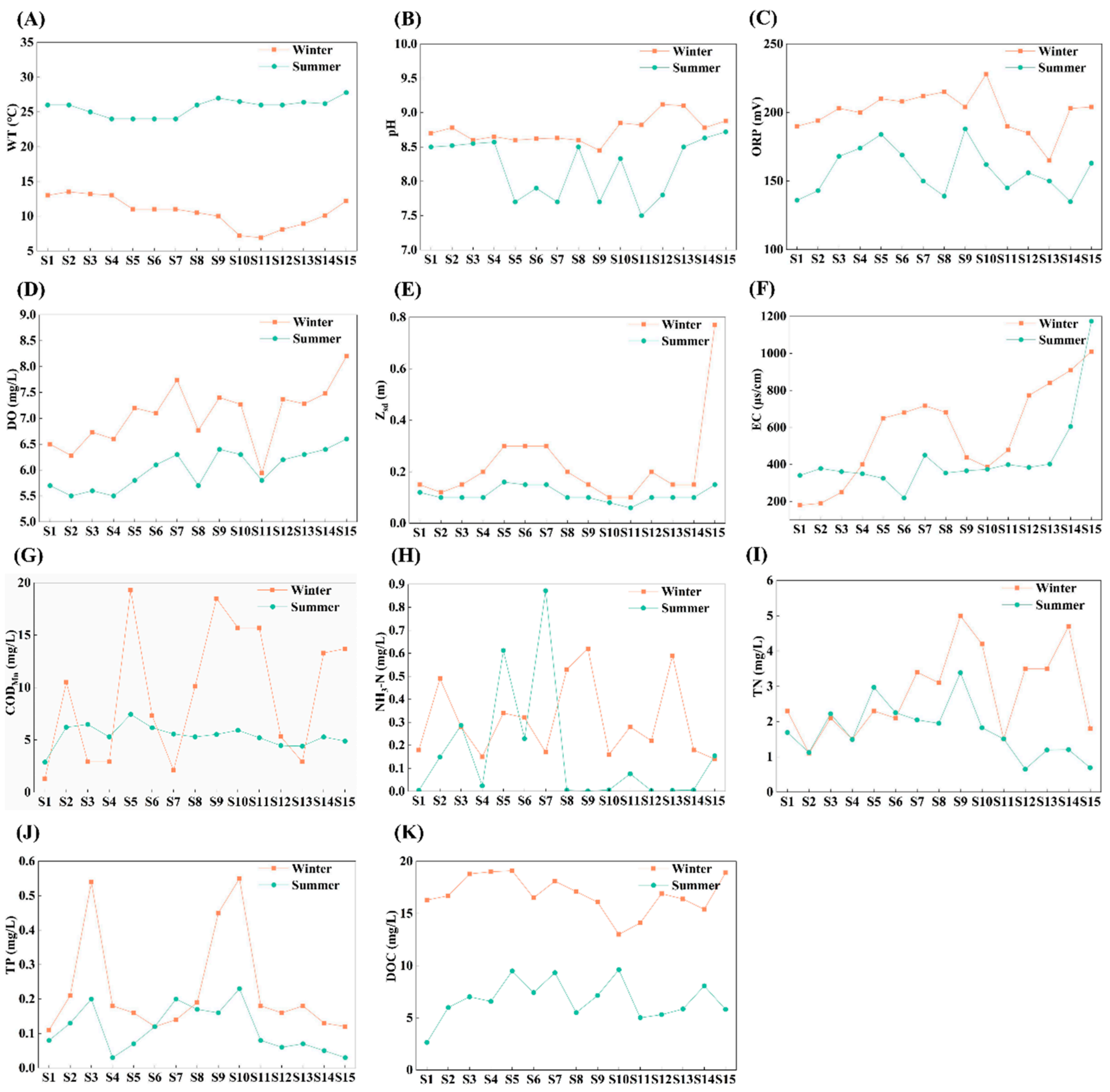


Figure S1. Physical and chemical parameters at each sampling point in winter and summer. (A) WT; (B) pH; (C) ORP; (D) DO; (E) Zsd; (F) EC; (G) $COD_{Mn}$; (H) $NH_3$-N; (I) TN; (J) TP; (K) DOC.

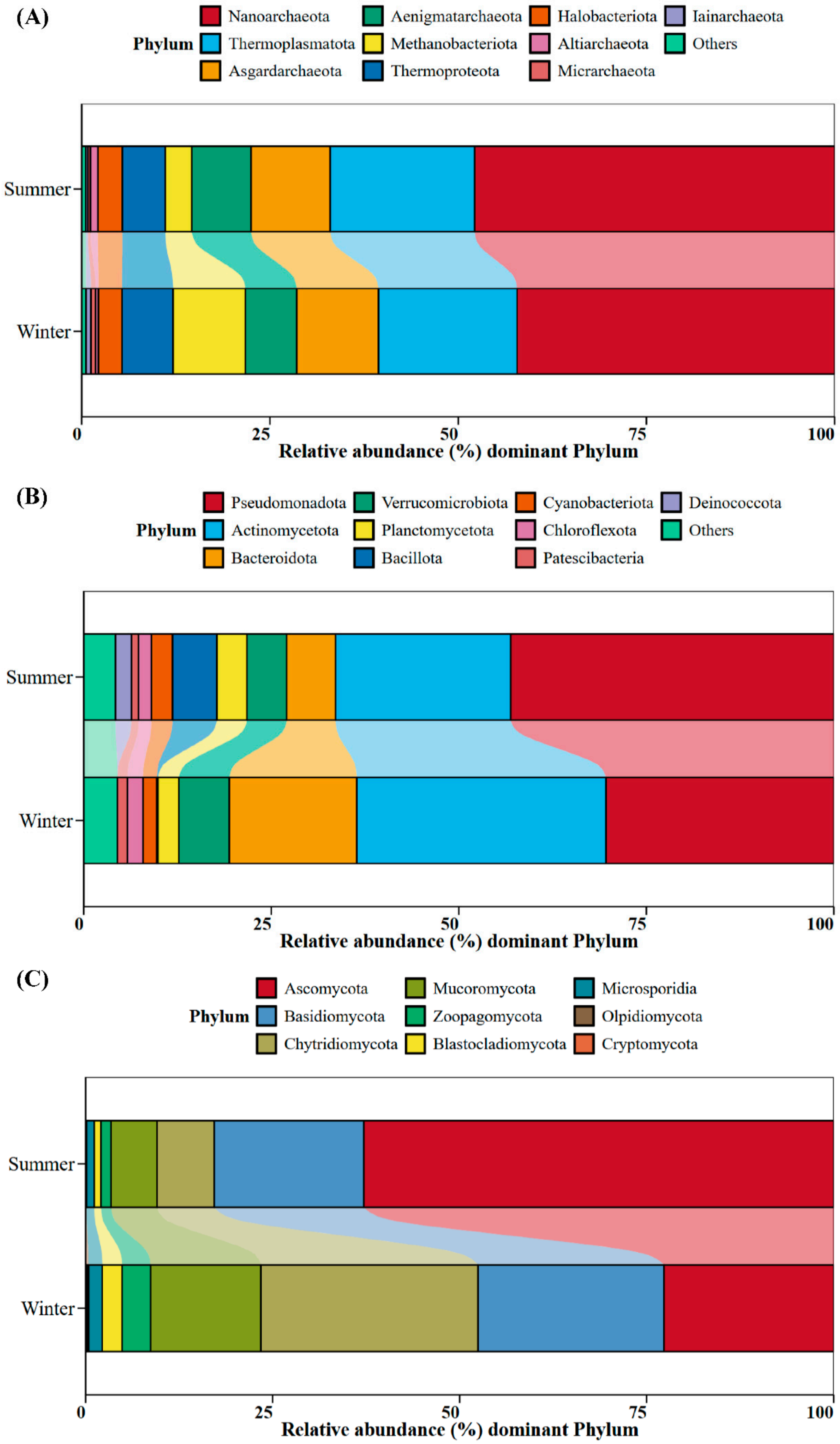
(A)
Phylum
Nanoarchaeota
Thermoplasmatota
Asgardarchaeota
Aenigmatarchaeota
Methanobacteriota
Thermoproteota
Halobacteriota
Altiarchaeota
Micrarchaeota
Iainarchaeota
Others
Summer
Winter
0
25
50
75
100
Relative abundance (%) dominant Phylum
(B)
Phylum
Pseudomonadota
Actinomycetota
Bacteroidota
Verrucomicrobiota
Planctomycetota
Bacillota
Cyanobacteriota
Chloroflexota
Patescibacteria
Deinococcota
Others
Summer
Winter
0
25
50
75
100
Relative abundance (%) dominant Phylum
(C)
Phylum
Ascomycota
Basidiomycota
Chytridiomycota
Mucoromycota
Zoopagomycota
Blastocladiomycota
Microsporidia
Olpidiomycota
Cryptomycota
Summer
Winter
0
25
50
75
100
Relative abundance (%) dominant Phylum

Figure S2. The relative abundance of the main phyla of (A) archaea, (B) bacteria and (C) fungi.

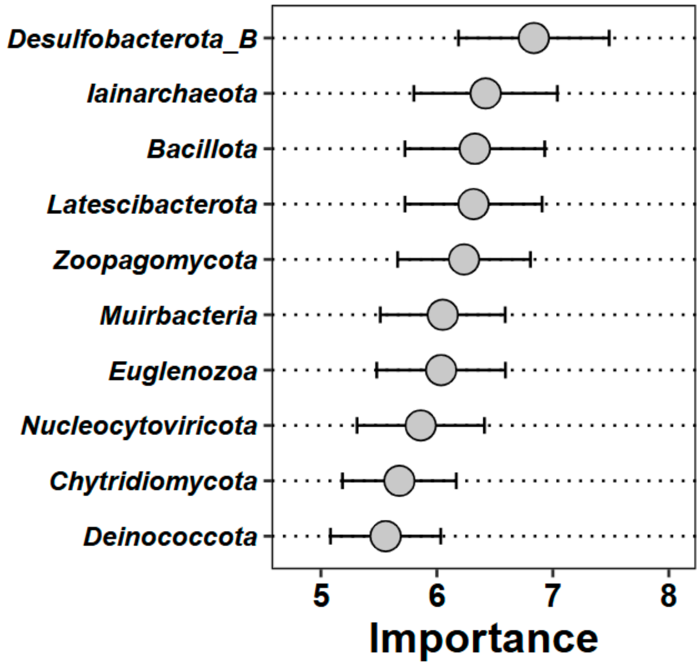


Figure S3. Random forest analysis (Total taxa - Phylum level).

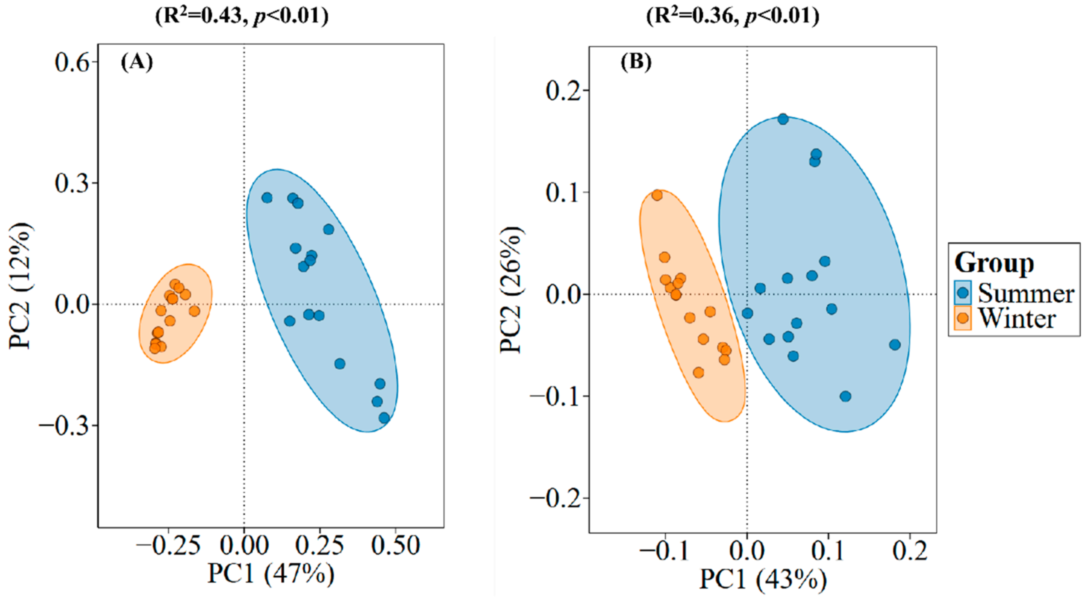


Figure S4. (A) Microbial communities and (B) functional gene PCoA analysis and Adonis test.

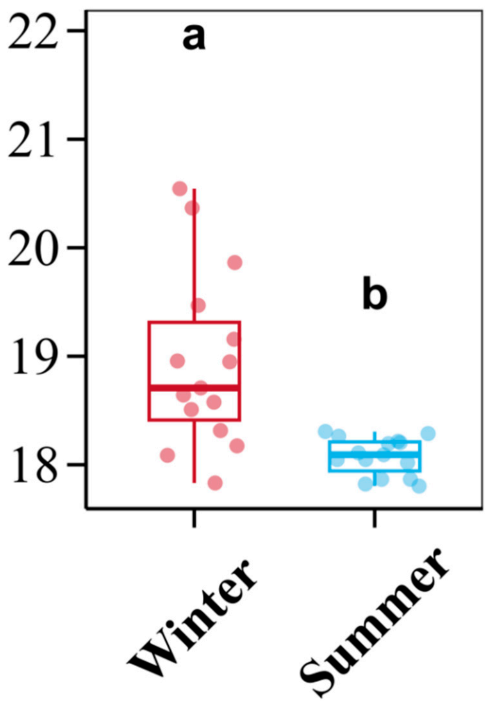

Figure S5. Niche breadth of functional genes.
